# Genome Sequences of Three *Oenococcus oeni* Strains Isolated from Maipo Valley, Chile

**DOI:** 10.1128/genomeA.00866-15

**Published:** 2015-08-13

**Authors:** Carla Jara, Jaime Romero

**Affiliations:** a Departamento de Agroindustrias y Enología, Facultad de Ciencias Agronómicas, Universidad de Chile, Santiago, Chile; b Laboratorio de Biotecnología, Instituto de Nutrición y Tecnología de los Alimentos, Universidad de Chile, Santiago, Chile

## Abstract

*Oenococcus oeni* is part of the microbial terroir involved in wine production. Here, we present three genome sequences of *O. oeni* strains isolated from spontaneous malolactic fermentation of cultivar Cabernet Sauvignon Maipo Valley, Chile.

## GENOME ANNOUNCEMENT

Winemaking is a complex process that involves two different fermentations, alcoholic fermentation and malolactic fermentation (MLF). MLF is an important stage impacting wine quality, in which lactic acid bacteria transform malic acid into lactic acid and CO_2_, decreasing the overall acidity of a wine and proving microbiological stability. In most Chilean wineries, the MLF stage of winemaking largely occurs in a spontaneous manner; thus, autochthonous *Oenococcus oeni* species are involved in this process (1). Chilean isolates might be autochthonous starter cultures for performing MLF in Cabernet Sauvignon grapes in Maipo Valley, Chile. The genome analyses might help understand the adaptation of the strains to wine-hostile conditions and their contribution to the organoleptic properties of the final product.

Bacterial genomes were sequenced using the Ion Torrent PGM platform with mate-paired end of 3-kbp span library for each isolate. The data were quality trimmed using Prinseq with a Phred score of 15, sequencing errors were corrected using the software Pollux, and data were subsequently assembled with Celera Assembler version 8.3. The genomic analysis was performed using the RAST server (2). The assembled sequence was annotated by the National Center for Biotechnology Information (NCBI) Prokaryotic Genomes Annotation Pipeline (PGAP). The genome information for each strain is summarized in Table 1. The genome size, G+C content, number of predicted genes, and number of RNA coding genes are comparable to those of the other published *O. oeni* strains (3, 4). These Chilean isolates showed genes related to the transformation of malic acid to lactic acid and citric acid metabolism. Furthermore, genes involved in biogenic amine formation (histamine and arginine) were not found; hence, they could be used as a safety starter for wines of a specific terroir, as suggested previously (5).

**TABLE 1:** Information for the whole genomes of three Chilean *O. oeni* strains

Strain	G+C content (%)	Genome size (bp)	No. of scaffolds	Accession no.	No. of tRNAs	No. of rRNAs (type)
139	38.06	1,949	32	LCTM00000000	46	12 (5S, 16S, 23S)
399	38.09	1,750	13	LCTP00000000	46	12 (5S, 16S, 23S)
565	37.71	1,755	16	LCTO00000000	45	12 (5S, 16S, 23S)

### Nucleotide sequence accession numbers.

These genome sequences were deposited in DDBJ/EMBL/GenBank under the accession numbers listed in Table 1. The versions described in this paper are the first versions of the assemblies. 
